# Neuron-to-neuron wild-type Tau protein transfer through a trans-synaptic mechanism: relevance to sporadic tauopathies

**DOI:** 10.1186/2051-5960-2-14

**Published:** 2014-01-30

**Authors:** Simon Dujardin, Katia Lécolle, Raphaëlle Caillierez, Séverine Bégard, Nadège Zommer, Cédrick Lachaud, Sébastien Carrier, Noëlle Dufour, Gwennaëlle Aurégan, Joris Winderickx, Philippe Hantraye, Nicole Déglon, Morvane Colin, Luc Buée

**Affiliations:** 1 Inserm, UMR837, Place de Verdun, 59045 Lille, France; 2 Faculté de Médecine, JPArc, Université Lille 2, Place de Verdun, 59045 Lille, France; 3 CMRR, CHR, 59037 Lille, France; 4 Atomic Energy Commission (CEA), Institute of Biomedical Imaging (I2BM), Molecular Imaging Research Center (MIRCen), F-92265 Fontenay-aux-Roses, France; 5 CNRS, URA2210, Molecular Imaging Research Center (MIRCen), F-92265 Fontenay-aux-Roses, France; 6 Functional Biology, KU Leuven, Kasteelpark Arenberg 31, box 2433, B-3001 Heverlee, Belgium; 7 Present address: Department of Clinical Neurosciences (DNC), Laboratory of Cellular and Molecular Neurotherapies (LMCN), Lausanne University Hospital (CHUV), CH-1011 Lausanne, Switzerland

**Keywords:** Alzheimer, Prion, Propagation, Secretion, spreading

## Abstract

**Background:**

In sporadic Tauopathies, neurofibrillary degeneration (NFD) is characterised by the intraneuronal aggregation of wild-type Tau proteins. In the human brain, the hierarchical pathways of this neurodegeneration have been well established in Alzheimer’s disease (AD) and other sporadic tauopathies such as argyrophilic grain disorder and progressive supranuclear palsy but the molecular and cellular mechanisms supporting this progression are yet not known. These pathways appear to be associated with the intercellular transmission of pathology, as recently suggested in Tau transgenic mice. However, these conclusions remain ill-defined due to a lack of toxicity data and difficulties associated with the use of mutant Tau.

**Results:**

Using a lentiviral-mediated rat model of hippocampal NFD, we demonstrated that wild-type human Tau protein is axonally transferred from ventral hippocampus neurons to connected secondary neurons even at distant brain areas such as olfactory and limbic systems indicating a trans-synaptic protein transfer. Using different immunological tools to follow phospho-Tau species, it was clear that Tau pathology generated using mutated Tau remains near the IS whereas it spreads much further using the wild-type one.

**Conclusion:**

Taken together, these results support a novel mechanism for Tau protein transfer compared to previous reports based on transgenic models with mutant cDNA. It also demonstrates that mutant Tau proteins are not suitable for the development of experimental models helpful to validate therapeutic intervention interfering with Tau spreading.

## Background

AD is the most common neurodegenerative disorder. It results from an accumulation of extracellular amyloid deposits and a neurodegenerative process called NFD, which is characterised by the intraneuronal aggregation of the microtubule-associated Tau proteins. This Tau pathology has been associated with a number of neurodegenerative disorders, referred to as tauopathies. In contrast to AD, where mutations have not been identified on the Tau gene (*MAPT*), patients presenting fronto-temporal dementia with parkinsonism, associated with chromosome 17 (FTDP-17), exhibit Tau mutations [1]. These mutations have been used to develop animal models with Tau aggregation and NFD to decipher the role of Tau in tauopathies [2,3]. However, the relevance of this approach remains unknown, as mutant Tau proteins show a higher nucleation process than WT Tau and fibrillogenesis, often leading to rapid neuronal death [4,5]. Moreover, in FTDP-17, there is no specific neural network affected by the Tau pathology. Conversely, certain sporadic tauopathies display hierarchical pathways of NFD. For example, in AD, neurodegeneration begins in the trans-entorhinal cortex, spreads to the hippocampal formation, anterior temporal cortex, and polymodal and unimodal association areas and eventually invades the entire cerebral cortex [6-8]. In progressive supranuclear palsy, another tauopathy, a specific pathway of NFD has also been identified leading from subcortical structures to the primary motor cortex through the *pedunculopontine* nucleus and eventually to other frontal regions [9]. The pathways of NFD have also been described for other tauopathies, such as argyrophilic grain disease, in which Tau aggregation starts in the vicinity of the ambient gyrus, then spreads to the temporal lobe and subiculum and entorhinal cortices and eventually reaches the septum, insular cortex and cingulate gyrus [10]. In addition to differences in these specific pathways, different Tau phosphorylation, isoforms, species and aggregates have also been identified among tauopathies [1]. Taken together, these characteristics are consistent with the presence of different Tau strains that might transfer Tau pathology from cell to cell [11].

Recent data suggest that Tau pathology may be induced and propagated after the injection of Tau oligomers and/or aggregates in either wild-type (WT) or mutated Tau transgenic mice [11-14]. Moreover, there is evidence that Tau aggregates can be transferred from cell to cell *in vitro*[15-19]. The hierarchical pathways of NFD in tauopathies might be associated with the trans-synaptic transfer of Tau pathology, as recently suggested *in vivo*[20,21]. However, these conclusions could be hampered by at least two factors: the use of a leaky inducible system that may induces the weak expression of the transgene in the hippocampus, and the use of mutated Tau protein to study propagation, as the spreading of Tau pathology is only observed in sporadic tauopathies, where no mutation on MAPT has been identified. In these models, Tau diffusion was consistently observed in close vicinity to the expression region, and no definite evidence of a direct cell-to-cell transfer of pathological Tau proteins was provided.

To address these two weaknesses, we took advantage of a recently developed lentiviral-mediated rat model of hippocampal NFD [22] to demonstrate that 1) WT Tau protein is transferred in a trans-synaptic manner from primary neurons located in the CA1 region of the hippocampal formation to many anatomically connected secondary neurons in different brain areas including the most distant ones (10 mm away from the injection site (IS)), 2) Tau species found in secondary connected neurons are mainly in a dephosphorylated form and 3) WT and mutated Tau species display differential spreading of Tau pathology.

## Methods

### Antibodies

Monoclonal antibody AT8 (Thermo Scientific MN1020 - 1:400 for immunolabelling) recognises phosphorylated residues serine 202 and threonine 205 of Tau, and monoclonal antibody AT100 (Thermo Scientific MN1060 - 1:400 for immunolabelling) recognises phosphorylated residues threonine 212 and serine 214 of Tau. Tau C-ter is a polyclonal rabbit antibody, which recognises the carboxyl terminal region of Tau [23]. The monoclonal antibody MC1 was a generous gift from Peter Davis and recognises conformational changes in residues seven to nine and 313-322 (1:1000 for immunolabelling). ADx215 is a human specific anti-Tau antibody that recognizes Tau only when Tyr18 residue is dephosphorylated [4]. Mouse monoclonal (Invitrogen P/N-0705; 1:10000 for immunolabelling) and rabbit polyclonal antibodies (1:10000 for immunolabelling) to V5 recognise the V5 epitope of tagged Tau.

### Plasmid construction

The packaging construct pCMVΔR8.92 was used. The Rev gene was inserted into the pRSV-Rev plasmid to minimise the risk of recombination and the production of replication-competent lentiviruses. Viral particles were pseudotyped with the vesicular stomatitis virus G-protein encoded in the previously described pMD2.G plasmid [24]. cDNAs encoding the 2 + 3-10+1N4R isoforms of human WT Tau and mutant P301L Tau were first cloned into the Gateway Entry pCR8/GW/TOPO vector (Invitrogen) using TOPO TA cloning methodology. The Gateway LR clonase (Invitrogen) catalysed the *in vitro* recombination between the Gateway Entry pCR8/GW/TOPO vector (containing the Tau cDNA flanked by attL sites) and the lentiviral destination vector (containing homologous attR sites). For specific constructs, the sequence of the epitope tag V5, previously validated *in vivo* using immunohistochemical analysis (14 aa, GKPIPNPLLGLDST) [25], was inserted into the cDNA encoding the 2+3-10+ isoform of the human WT Tau between the sequences encoding exons two and four.

### Production and assay of recombinant lentiviral vectors (LVs)

LVs vectors were amplified as previously described [24] and encode either for V5-hTau46^WT^, hTau46^WT^, hTau^P301L^ or eGFP proteins. Human 293 T cells (4 × 10^6^) were plated onto 10-cm plates and transfected the following day with 13 μg of human Tau cDNA, 13 μg of pCMVΔR8.92, 3 μg of pRSV-Rev and 3.75 μg of pMD.2G using the calcium phosphate DNA precipitation procedure. Four to six hours later, the medium was removed and replaced with fresh medium. Forty-eight hours later, the supernatant was collected and filtered. High-titre stocks were obtained through two successive ultracentrifugation steps at 19,000 rpm (Beckman Coulter SW 32Ti and SW 60Ti rotors) and 4°C. The pellet was resuspended in PBS with 1% bovine serum albumin (BSA) and stored frozen at -80°C until further use. Viral concentrations were determined through ELISA for the HIV-1 p24 antigen (Gentaur BVBA). The p24 protein is a lentiviral capsid protein that is commonly used in ELISA assays to determine the physical titre of lentiviral batches per ml. All viral batches were produced in appropriate areas in compliance with institutional protocols for genetically modified organisms according to the ‘Comité Scientifique du Haut Conseil des Biotechnologies’ (Identification Number 5258).

### Neuronal cultures, microfluidic chamber system and assays for the neuron-to-cell transfer of Tau

Briefly, glass coverslips were coated overnight at 4°C with 0.5 mg/ml of poly-D-Lysine (SIGMA). The microfluidic chamber (AXIS™, Temecula, CA) was subsequently placed on coated glass coverslips and sealed to the glass. Rat Primary embryonic neuronal cultures were performed as previously described [26], and approximately 30,000 cells each were plated in the two wells of the somatodendritic compartment. The cultures were maintained at 37°C for 15 days for differentiation. All chambers had microgrooves of 450 µm in length and 10µm in width. One week post-plating, after axonal growth across the microgrooves, a second rat primary embryonic neuronal culture were plated in the axonal compartment in the appropriate medium. Twenty-four hours later, the V5-Tau-LVs or eGFP-LVs (200 ng of LVs per well) were added to the somatodendritic compartment after first reversing the volume gradient between the compartments to counteract viral diffusion. Forty-eight hours later, eGFP fluorescence and V5 immunolabelling were analyzed. The compartments (somatodendritic and axonal) were washed once with phosphate-buffered saline (PBS) and fixed with 4% paraformaldehyde (PFA) for 20 min. After removing the fixative, the cells were washed three more times with 50 mmol/L NH_4_Cl and processed as described in the immunofluorescence section.

### Testing of fluidic isolation

Isolation of the different compartments in microfluidic conditions without cells was also assessed using either Coomassie Blue or LVs particles. Coomassie Blue (2%) or LVs particles (400 ng p24) diluted in PBS were added to the somatodendritic compartment of microfluidic device and PBS was added to the axonal compartment. The volume gradient between both compartments was adapted to avoid or to mediate diffusion across the microgrooves. 5 min, 1, 2, 24 or 48 h later, optical density at 595 nm was measured.

When LVs particles were used instead of Coomassie Blue, the medium in both compartments was recovered and viral RNA extracted (Nucleospin RNA Virus, MACHEREY-Nagel, Düren, Germany). cDNAs were generated by RT-PCR and PCR done using the following oligonucleotides specific to the viral WPRE (Woodchuck Hepatitis Post-transcriptional Regulatory Element) (forward: 5′-TAC-GCT-ATG-TGG-ATA-CGC-TGC-3′ and reverse: 5′-AAT-TCC-CGA-TGC-GGG-GA-3′).

### Animals

The animals were purchased from Janvier Laboratories and housed in a temperature-controlled room maintained on a 12 h day/night cycle with food and water provided *ad libitum*. The present experimental research has been performed with the approval of an ethics committee (‘Comité d’éthique en expérimentation animale du Nord Pas-de-Calais’-CEEA 342012) and follows internationally recognized guidelines.

### Stereotaxic injections and sacrifice procedures

Intracerebral injections of viral particles into the brain of anesthetised 2-month-old Wistar rats (Ketamine 100 mg/kg, Xylazine 10 mg/kg i.p.) were performed using classic stereotaxic procedures at the following coordinates relative to bregma: posterior, -5.3 mm; lateral, +/- 6.2 mm; ventral, -7 mm and -6.2 mm; depth. The injections were performed bilaterally. The standard injection procedure consisted of the delivery of 400 ng of p24 using a 10 μL glass syringe with a fixed needle (Hamilton). After injection at a rate of 0.25 μl per min, the needle was left in place for 1 min before reaching the second depth; the second injection was performed after 5 min. Control groups (n = nine) consisted of rats injected with PBS instead of LVs. For anterograde tracing, 5% biotinylated dextran amines (molecular weight 10 000 kilodaltons (kD); BDA 10 000, Invitrogen) were injected using 10 μl glass syringes with fixed needles (Hamilton).

*For immunohistochemical analyses*, animals were anesthetised (8% chloride hydrate) at two (n = 13), four (n = 13) or eight (n = 13) months post-injection and transcardially perfused first with cold 0.9% NaCl followed by 4% PFA for 20 min before beheading. The brains were immediately removed, fixed overnight in 4% PFA, placed in 20% sucrose for one week and frozen until further use. Free-floating coronal cryostat sections (40 μm thickness) were used for immunohistochemical analysis.

*For RNA extraction*, rats (n = eight) were deeply anesthetised using 8% chloride hydrate. Brains were dissected, and 1-mm-thick coronal sections were generated using an acrylic rat brain matrix (Electron Microscopy Sciences). The sections were immediately frozen on dry ice and stored at -80°C until further use.

*For anterograde transport studies*, rats were sacrificed 1 week post-dextran-injection and transcardially perfused with 0.9% NaCl and 4% PFA in 0.1 mol/L phosphate-buffered saline (pH 7.4). Brains were post-fixed for 24 hours in 4% PFA and cryoprotected before freezing for storage. Coronal sections (40 μm thickness) were washed three times in 0.1 mol/L PBS containing 0.2% Triton X-100 and incubated with fluorescein streptavidin (Vector) for 1 hour. After three washes, sections were mounted onto gelatine-coated slides and coverslipped with Vectashield Mounting Medium (Vector).

### Immunohistochemistry

The sections from the entire brain were washed in PBS-0.2% Triton and treated for 30 min with H_2_O_2_ (0.3%). Non-specific binding was then blocked using goat serum (1:100 in PBS, Vector) for 60 min. Incubation with the primary antibody in PBS-0.2% Triton was performed overnight at 4°C. After several washes, labelling was amplified by incubation with an anti-mouse biotinylated IgG (1:400 in PBS-0.2% Triton, Vector) for 60 min followed by the application of the ABC kit (1:400 in PBS, Vector) prior to visualisation with 0.5 mg/ml DAB (Vector) in Tris-HCl 50 mmol/L, pH 7.6, containing 0.075% H_2_O_2_. Brain sections were mounted onto gelatine-coated slides, stained for 1 min in a cresyl violet solution (0.5%), washed in water with 2% acetic acid, dehydrated by passage through a graded series of alcohol and toluene and mounted with Vectamount (Vector) for microscopic analysis.

### Immunofluorescence

*For brain sections*: sections from the entire brain were washed in PBS-0.2% Triton and blocked with goat serum (1:100 in PBS, Vector) for 60 min. Incubation with a primary antibody in PBS-0.2% Triton was performed overnight at 4°C. After several washes, the primary antibody against the second antigen was added in PBS-0.2% Triton, and the sections were again incubated at 4°C overnight. Incubation with the two Alexa Fluor secondary antibodies (1:1000 in PBS) was performed for 60 min at room temperature. The slides were mounted with Vectashield containing 4′,6-diamidino-2-phenylindole (DAPI) to label the nuclei (Vector). *For cell cultures*: sections were rinsed once in 50 mmol/L NH_4_Cl, and cells were permeabilised with Triton X-100 (0.1%, 10 min at room temperature). Subsequently, the slides were incubated with primary antibodies at 4°C overnight, and labelling was performed by a reaction with the appropriate Alexa Fluor secondary antibodies (1:400-Invitrogen) for 45 min at room temperature. The cells were mounted with Vectashield medium containing DAPI (Vector, Burlingame, USA).

### Fluorescence imaging

Confocal microscopy was performed using a Zeiss LSM 710 inverted confocal microscope. For each optical section, two fluorescence images were obtained. The signal was subjected to line averaging to integrate the signal collected over two or four lines to reduce noise. The confocal pinhole was adjusted to facilitate a minimum field depth. A focal series was collected for each specimen. The focal step between sections was typically 1 μm.

### RNA extraction from brain sections and RT-PCR/PCR

RT-PCR of lentiviral mRNA was performed using total RNA. The brain slices were lysed, and total RNA was extracted using the RNeasy Lipid Tissue kit (Qiagen, France) according to the manufacturer’s instructions. The RNA (1 μg) was denatured for 10 min at 68°C, and cDNA was generated using reverse transcription with 200 nmol/L of dNTPs, 1 ng/μl of random primers, 1 ng/μl of oligo dT, 5 mmol/L of dithiothreitol (DTT), 2 units/μl of RNase Out and 10 units/μl of M-MLV reverse transcriptase. The viral cDNAs were then amplified using oligonucleotides specific to human Tau (forward: 5′-TGG-GGG-ACA-GGA-AAG-A-3′ and reverse: 5′-CCT-CAG-ATC-CGT-CCT-CAG-TG-3′). The following pair of primers was used to amplify murine Tau for calibration: forward: 5′-CAC-AAT-GGA-AGA-CCA-GGC-C-3′ and reverse: 5′-TAA-GCC-ATG-GCT-CAT-GTC-TCC-3′. PCR was performed using 2 μl of the previously obtained RT products, reverse and forward primers (0.5 μmol/L), dNTPs (1 μmol/L) and 0.02 unit/μl of DNA polymerase in a commercial reaction buffer (GoTaq Green Master Mix, Promega). The PCR products were electrophoresed on an 8% acrylamide gel stained with 1 μg/ml ethidium bromide.

## Results

### A mechanism of trans-synaptic transfer of WT Tau protein can be demonstrated in vitro

We first determined whether the human WT Tau protein could be transferred between neuronal cells. Using a microfluidic device [27-29] comprising two compartments connected through embedded microgrooves, we studied the potential transfer of WT Tau from the somatodendritic compartment seeded with rat primary neurons to an axonal compartment seeded with a second rat primary embryonic neuronal culture. Microfluidic devices have already been validated for the axonal transport of recombinant α-synuclein or Tau [29,30]. To address this question in our LVs assay and to characterise the human Tau protein, we designed a new LV encoding a WT Tau with a V5 epitope (See Additional file 1). First of all, we performed several assays to control that LVs are not able to diffuse in the microgroove or to traffic along the microtubule (See Additional file 2).

**Figure 1:**
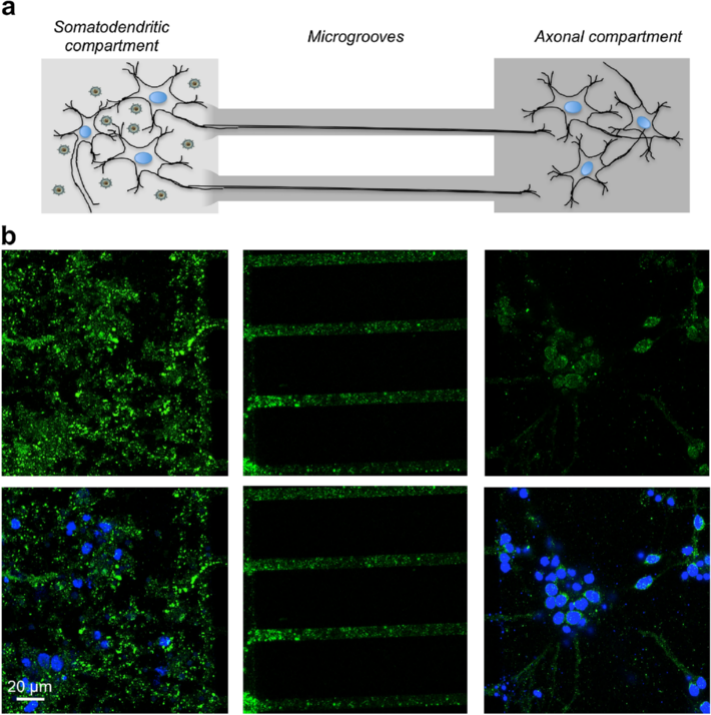
**Neuron-to-cell spread of WT Tau in a microfluidic device.** The microfluidic device used in our study comprised two compartments separated by a physical barrier containing microgrooves that facilitate the passage of axons, but not neuronal cell bodies, during neuronal differentiation. **(a)** Primary culture of embryonic rat cortical neurons seeded in the first compartment (somatodendritic) was infected at DIV 7 with LVs encoding V5-hTau46^WT^. The flow was then reversed and a second rat primary embryonic neuronal culture cells was seeded in the axonal compartment. **(b)** Forty-eight hours post-infection, the cells were processed for immunofluorescence analysis using anti-V5 antibodies and an Alexa Fluor 488-labeled secondary antibody (green). The nuclei were counterstained with DAPI (blue). The scale bar is indicated on the figure. These data showed that V5 is found in axons in primary neurons and in cell bodies of secondary neurons in the axonal compartment.

After differentiation of rat brain primary neuronal cultures [27], the hydrostatic pressure difference was reversed to avoid viral vector diffusion, and an LV was added to the somatodendritic compartment to facilitate the expression of the WT V5-Tau in rat primary neurons. Simultaneously, a second rat primary embryonic neuronal culture was seeded in the axonal compartment. After 48 hours, V5-immunoreactivity was primarily observed in the somatodendritic compartment and in axons located in the microgrooves. Interestingly, V5 immunoreactivity was also detected in secondary neurons, indicating the cell-to-cell transfer of WT V5-Tau protein via axonal transport from the primary neurons (Figure 1). This transfer was specific to Tau since GFP was not found in secondary neurons (See Additional file 2). Finally, all controls in the axonal compartment were negative. It is not surprising since, due to the speed of HIV axonal transport and its time of uncoating, LVs are not able to reach the axonal endings in the present microfluidic devices where the length of microgrooves is 450 μm [31,32]. These results demonstrate *in vitro* that donor neurons overexpress human WT V5-Tau, which is transported along axons, secreted and taken up by acceptor cells.

### A mechanism of trans-synaptic transfer of WT Tau protein can be demonstrated in vivo

**Figure 2:**
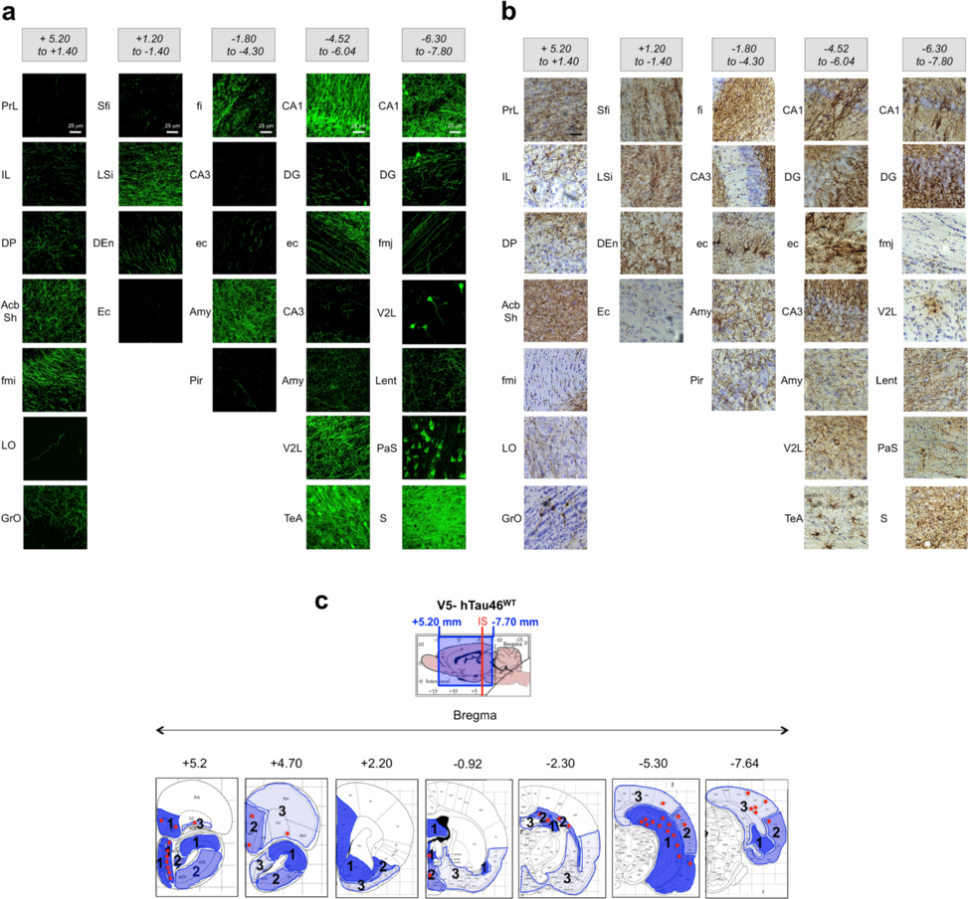
**The transfer of V5-hTau46**^**WT **^**protein is correlated to brain area connected to the IS (caudal part of the CA1 layer). (a)** Dextran amines (10 kD) are anterograde tracers: they were injected into the rat brain (n = 3), and one week later, the animals were sacrificed and their brains were processed to reveal fluorescent axons efferent from the IS. Brains were virtually divided into five sections: bregma +5.20 to +1.40, bregma +1.20 to -1.40, bregma -1.80 to -4.30, bregma -4.52 to -6.04 and bregma -6.30 to -7.80. **(b)** LVs encoding V5-hTau46^WT^ were bilaterally injected into the CA1 layer (IS; bregma -5.3) of rat brains (n = 3). Five months later, the animals were sacrificed, and the whole brain was processed for immunohistochemical analysis using a rabbit polyclonal anti-V5 antibody. The brains were virtually divided into five sections as in **(a)**. The bar scale is indicated on the figure. The drawing showing the extension of WT V5-Tau protein transfer from the IS to extreme rostral and caudal positions is illustrated in the lower part of the figure. These data showed that V5-hTau46^WT^ Tau is transported throughout the brain using neural networks. **(c)** The V5-immunoreactivity is summarized in a cartoon drawing of several coronal sections at different bregma coordinates. The different blue intensities (level 1 to 3) indicate the density of fibres and the red stars indicate the presence of V5-immunopositive cellular bodies indicating a trans-cellular transfer of V5-hTau46^WT^ Tau.

The first aim of this study was to identify the IS in the rat brain that would facilitate not only the monitoring of cell-to-cell transfer of Tau protein in distant brain areas but also to discriminate between free protein diffusion and active transfer through anatomically organised neuronal pathways. Using published data on the rat brain neuroanatomy [33], we selected stereotactic coordinates in the caudal region of the CA1 layer, which is associated with brain regions remote from the injected area and is anatomically connected through well-defined neural networks. First, we assessed these regions using an anterograde tracer [34], and as expected, these areas were identified among projecting fibres throughout the brain, i.e., those in rostral areas, such as limbic regions, olfactory/orbital systems and caudal areas, such as the subiculum and entorhinal cortex (Figure 2a). Using the LV encoding WT V5-Tau protein, we injected rats in the CA1 region of the hippocampus. Five months post-injection, V5-immunohistochemistry was performed and V5-immunoreactivity was found in different regions of the whole brain (Figure 2b). More importantly, both axonal tracer labelling and V5-were found associated with the same regional pattern supporting that WT Tau protein is transferred in each area connected to the IS (see Figure 2a and b). Most of the V5-immunoreactive cell bodies were observed in several connected regions in caudal but also in rostral brain areas (Figure 2c). For instance, some cell bodies were found as far as 10 mm from the IS in the granular layer of the olfactive bulb (GrO, Bregma +5.2). Altogether, these analyses strongly suggest that the spatio-temporal transfer of WT Tau is progressing through neural networks.

**Figure 3:**
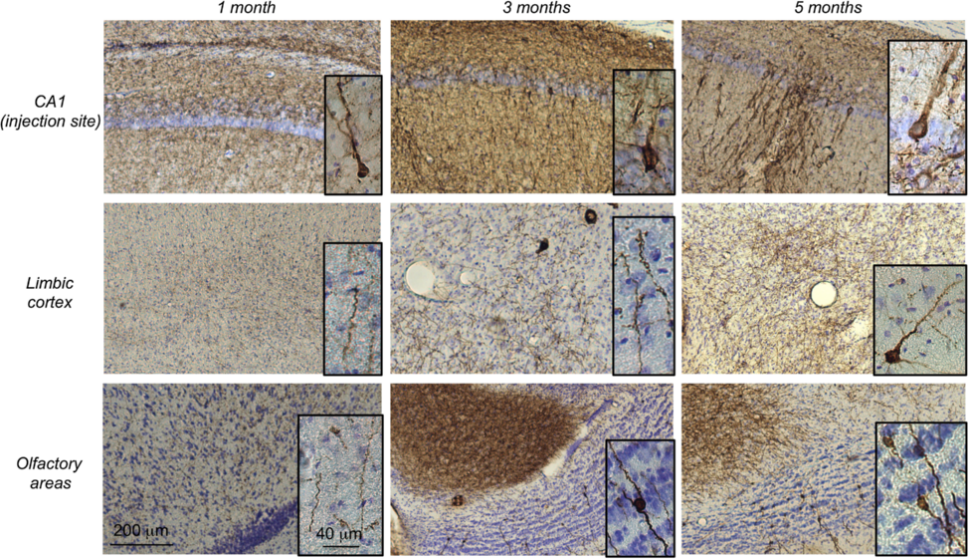
***In vivo *trans-synaptic transfer of WT Tau protein.** LVs encoding V5-hTau46^WT^ were bilaterally injected into the CA1 layer (IS; bregma -5.3) of rat brains (n = 3 rats per group). One, three and five months later, the animals were sacrificed, and the whole brains were processed for immunohistochemical analysis using an antibody to total V5-Tau. Sections from the prelimbic or orbital cortex (bregma +4.7), the olfactory bulb (bregma +5.2) and the CA1 (bregma -5.3, IS) are shown. The scale bars are indicated on the figure. These data showed that V5-hTau46^WT^ is transported from primary to secondary neurons in a time-dependent manner.

**Figure 4:**
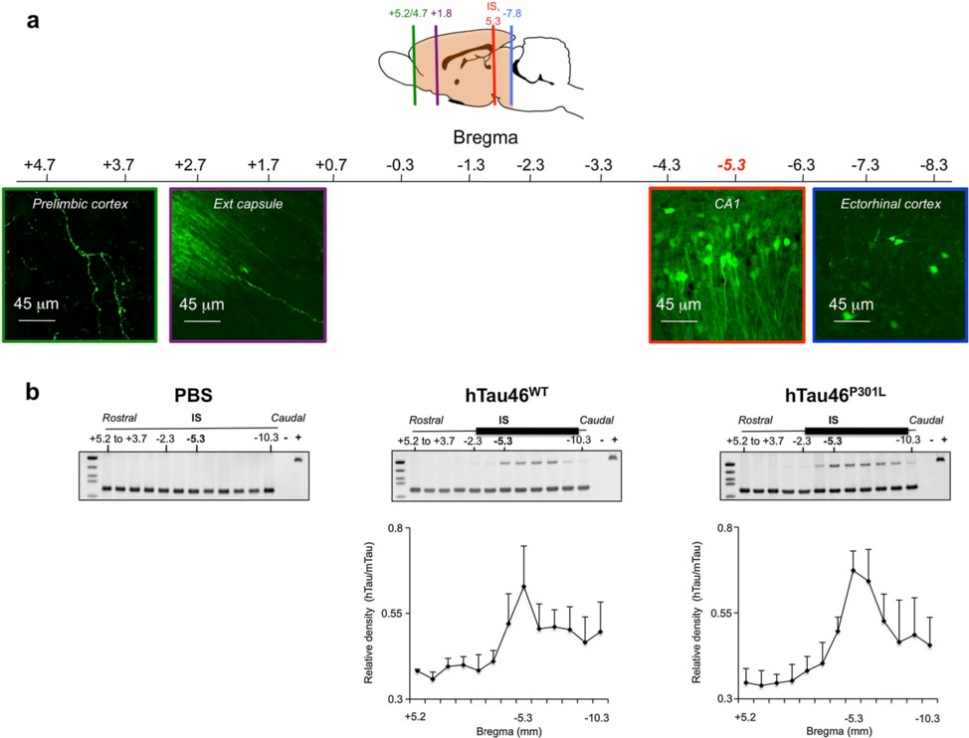
**Cell-to-cell protein transfer is specific to Tau protein WT. (a)** eGFP is not transported in secondary connected neurons. LVs encoding eGFP were bilaterally injected into the CA1 layer of rat brains (IS bregma -5.3, n = 3). Eight months later, the animals were sacrificed, and brains sections processed for immunofluorescence assays to detect eGFP. Sections from the prelimbic (bregma +4.7), external capsule (bregma -1.8), CA1 (IS, bregma -5.3) and ectorhinal cortex (bregma -7.8) are shown. The scale bars are indicated on the figure. **(b)** Restricted lentiviral insertion in the vicinity of the IS. PBS (left panel, n = 3) or LVs encoding hTau46^WT^ (middle panel, n = 3) or hTau46^P301L^ (right panel, n = 3) were bilaterally injected into the CA1 layer of rat brains. One month later, the brains were dissected, and coronal sections of 1 mm thickness (indicated as the position relative to bregma) were prepared using an acrylic rat brain matrix. Total RNA was extracted from these sections to generate cDNA using RT-PCR. cDNAs were amplified using oligonucleotides specific to human Tau (hTau, 117 base pairs) or murine Tau (70 base pairs). The positive control was prepared by amplifying the plasmid containing hTau sequence. The lower parts represent the mean +/-SEM of the relative density (hTau/mTau) coming from the three rats. These data showed that Tau transport is a specific mechanism since neither eGFP nor viral genome is found associated to distant secondary brain areas.

A follow-up study of V5-immunoreactivity in the rat brain was also performed at 1, 3 and 5 months post-injection to assess the time-dependent distribution of the tagged WT human Tau protein. As an example, we focused on two of the most rostral distant systems: the olfactory and the limbic areas. At 1 month post-injection, V5-immunoreactivity was observed at the IS and in fibres projecting in both areas. No labelled neuronal cell bodies were observed at this stage. At 3 months post-injection, in addition to fibres, some V5-immunoreactive cell bodies (50 < cell bodies < 100, N = 3 rats, bregma +5.2) were visualized in the olfactory area, whereas more fibres, but no cell bodies, were observed in the limbic cortex. At five months post-injection, numerous V5-immunoreactive cell bodies were observed mainly in the olfactory area (cell bodies > 100, N = 3 rats, bregma +5.2) but also in the limbic regions (5 < cell bodies < 10, N = 3 rats, bregma +5.2) (Figure 3). Such dynamic process over time supports that WT Tau is transported through axons and then trans-synaptically transferred to secondary neurons in different brain regions. To further demonstrate that this WT Tau transport results from an intrinsic property of the Tau species and not from our model used to induce local overexpression of the transgene i.e. the LVs, different controls were used. Indeed, one explanation for the occurrence of Tau proteins far from the IS could be the diffusion of LVs during the injection process, although this is unlikely to be the case since 1) LVs delivery remains spatially restricted to the injected area when a reporter green fluorescent protein (GFP) is used instead of WT V5-Tau protein. Indeed, GFP protein was observed only in neurites, never in secondary neurons in connected areas (ie: the prelimbic structures and olfactory bulb) (Figure 4a) and 2) human Tau RNA was never detected in areas distant from the IS (Figure 4b). Polymerase chain reaction was used to detect human Tau RNA in sections covering the entire brain. The presence of the human Tau RNA was restricted to the area around the IS, where it followed a Gaussian distribution for both forms of Tau. It was neither detected in PBS-injected rats nor in areas distant from the IS in LVs-injected animals, whereas V5 immunoreactivity in WT V5-LVs-injected rats was found in cell bodies in connected secondary regions (Figure 2b), supporting that the mechanism of cell-to-cell protein transfer is likely specific to WT Tau protein.

We therefore concluded that WT V5-Tau protein, expressed in CA1 neurons, can be 1) transported through normal CA1-efferent projections even to distant brain regions, such as the olfactory and limbic systems and 2) secreted and transferred intercellularly to secondary neurons located in CA1 efferent regions.

**Figure 5:**
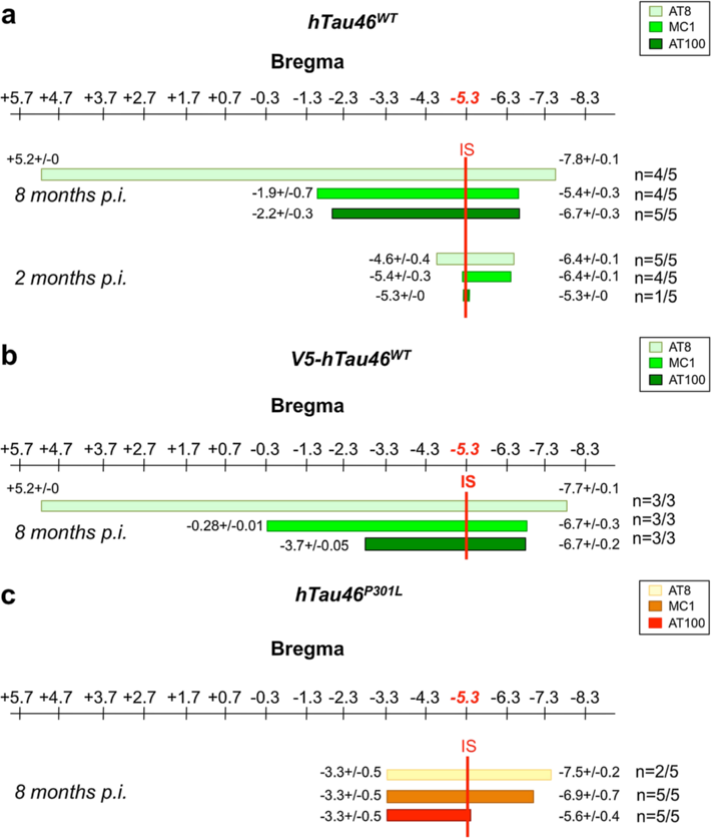
**Differential Tau spreading between WT and mutated Tau species.** LVs encoding hTau46^WT^ (n = 5, sacrificed at 2 months; n = 5, sacrificed at 8 months) **(a)**, V5-hTau46^WT^ (n = 3, sacrificed at 8 months) **(b)** or hTau46^P301L^ (n = 5, sacrificed at 8 months) **(c)** were bilaterally injected into the CA1 layer of rat brains. After sacrifice, the whole brain was processed for immunohistochemical analysis using AT8, MC1 or AT100 phosphorylated Tau antibodies. Among the positive rats, the rostralmost and caudalmost brain coordinates (from bregma) labeled by each antibody were determined for each brain. The figures represent the mean values +/-SEM of the rostralmost of caudalmot brain coordinates. These data showed that whereas hTau46^P301L^ diffusion is restricted to the vicinity of the IS, hTau46^WT^ has spread throughout the brain. Numbers of immunopositive rats per group are indicated on the right of each mapping.

To confirm that the V5 tag does not affect the behaviour of Tau proteins in the trans-synaptic transfer, we compared the observed effects to that of non-tagged Tau proteins. The progression of Tau pathology was followed using anti-Tau antibodies specific for different pathological states of the Tau protein: a hyperphosphorylated state (AT8 [35]), a conformational change (MC1 [36,37]) and an aggregated form (AT100 [38]) already validated in this model [22]. Antibody mappings of the whole rat brain showed differential gradients of Tau pathology spreading (Figure 5). These data also supports that the V5 tag does not interfere with the progression Tau pathology throughout the brain (compared Figure 5a and b). More interestingly, in a two points kinetic study, we observed that two months post-injection, Tau pathology related to WT Tau was only weakly detected at the IS, as described by Caillierez et al. [22] whereas the extended spreading of the Tau pathology was observed throughout the brain 8 months post-injection (Figure 5a).

**Figure 6:**
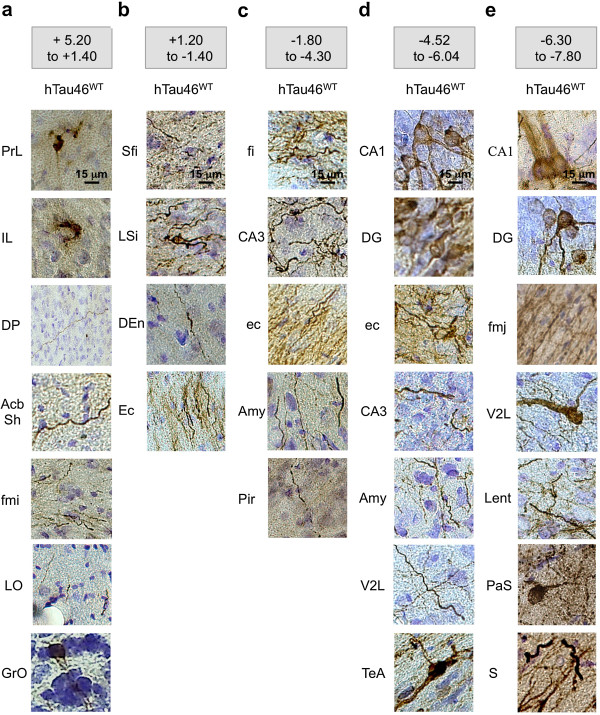
**Spatiotemporal progression of the Tau pathology through neural networks.** LVs encoding hTau46^WT^ were bilaterally injected into the CA1 layer of rat brains (n = 5). Eight months later, the animals were sacrificed, and the whole brain was processed for immunohistochemical analysis to show AT8-related pTau. The brains were virtually separated into five sections: **(a)** bregma +5.20 to +1.40, **(b)** bregma +1.20 to -1.40, **(c)** bregma -1.80 to -4.30, **(d)** bregma -4.52 to -6.04 and **(e)** bregma -6.30 to -7.80. The bar scale is indicated on the figure. These data showed that phospho-hTau46^WT^ is found all over the brain eight months post-LVs delivery.

To determine the influence of Tau species in the propagation process, we then compared Tau pathology induced by non-tagged Tau proteins with or without a P301L mutation (Figure 5c). P301L exhibited a severe Tau pathology (primarily immunoreactive for all antibodies) with a narrow diffusion (up to 2 mm from the IS on both the rostral and caudal sides). Conversely, WT Tau showed Tau pathology with a wider spatial propagation in both the rostral (10 mm ahead of the IS with AT8-immunoreactivity; 4 mm ahead with MC1-immunoreactivity) and caudal (greater than 2 mm from the IS) CA1-projecting regions (Figure 5a, Additional files 3 and 4). The pathological spreading clearly progressed through the brain neural networks, as evidenced by the co-localisation between phosphorylated Tau (pTau) and anterograde tracer as well as the lack of pTau in dextran-negative regions (Figure 2a and 6).

### Transferred Tau species are mainly in a dephosphorylated state

**Figure 7:**
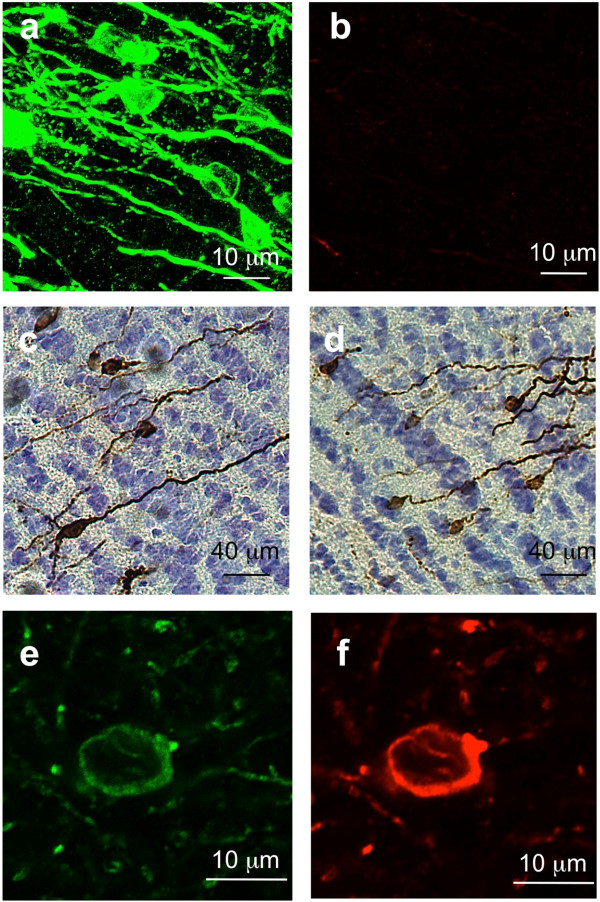
**Transferred Tau species are mainly in a dephosphorylated state.** LVs encoding V5-hTau46^WT^ were bilaterally injected into the CA1 layer (IS; bregma -5.3) of rat brains (n = 3). Five months later, the animals were sacrificed, and the rostral areas of the brain were processed for immunofluorescence analysis using a rabbit polyclonal anti-V5 antibody to detect total Tau and a mouse monoclonal antibody to pTau (AT8). The V5-Tau proteins were visualised in green using the corresponding secondary antibody Alexa Fluor-488 labelled goat anti-rabbit IgG **(a and e)** and the phospho-Tau in red using Alexa Fluor-568 labelled goat anti-mouse IgG **(b and f)**. The scale bars are indicated on the figure. Adjacent brain slides from the rostral areas were assessed by immunohistochemistry using an antibody directed against the N-terminal portion of Tau containing a dephosphorylated tyrosine 18 residue (ADx215) **(c)** or an antibody to total V5-Tau **(d)**. Most of hTau46WT Tau species found in secondary neurons are dephosphorylated.

It remains unknown whether the AT8-immunoreactivity observed in distant brain areas is associated with endogenous rat Tau or human Tau. To address this question, we performed additional co-labeling assays using WT V5-Tau five months post-injection. In distant brain regions, the majority of the V5-immunoreactive neurons was not AT8-immunoreactive (Figure 7a and b) suggesting that serine residues 202 and 205 are dephosphorylated. We also tested the phosphorylation state of tyrosine 18 using additional antibody, ADx215, which recognized Tau only when this residue is dephosphorylated (ADx215 [4]). The immunoreactivities of both ADx215 (Figure 7c) and V5 (Figure 7d) look like similar; suggesting that most Tau associated to secondary neurons in the GrO is dephosphorylated on tyrosine 18. More interestingly, a few secondary neurons displayed both AT8 and V5 immunoreactivities (7%), indicating the hyperphosphorylation of the transferred human V5-Tau protein (Figure 7e and f). It should be noted that less than 1% of the secondary neurons in the olfactory system were AT8-immunoreactive without any V5 labelling suggesting a prion-like conversion process. Altogether, these data suggest that only a few pathological Tau species are present in secondary neurons consistent with the slow kinetics of Tau spreading in sporadic Tauopathies.

## Discussion

In the present study, we demonstrated *in vivo* that WT Tau proteins are transferred through primary neurons from the IS to secondary neurons in distant rat brain areas to initiate Tau pathology. Recent studies have shown that the Tau pathology might spread *in vivo* through mono-synaptic connections in the hippocampal formation [20,21]. Nevertheless, the transfer of Tau proteins from neuron to neuron has not been conclusively demonstrated. For example, hippocampal NFD might result from either trans-synaptic transfer or signal transduction associated with neuronal processes, such as receptor activation through Tau aggregation/oligomerisation [39,40].

*In vitro*, there is now considerable evidence demonstrating that Tau is secreted into the extracellular medium [15,17,18,30,41-44] and that Tau aggregates are internalised in different cell lines [15-19,41,45,46]. Nevertheless, to date there has been no evidence of trans-synaptic protein transfer.

*In vivo*, Tau aggregates from P301S transgenic mice and human brains, injected into Alz17 mice (mouse strain overexpressing the longest Tau isoform), were captured/internalised by neurons at the IS. Some degree of diffusion was observed at the vicinity of the IS, reminiscent of Tau spreading [11,12]. In contrast, an inducible mouse line, in which the tTA activator is driven through a neuropsin promoter specific for the entorhinal cortex, was cross-bred with the tetracycline-inducible Tau Tg mouse line, rTg4510, expressing human four-repeat Tau with the P301L mutation. In the resulting rTgTauEC mice, neurons expressing P301L Tau species in the trans-entorhinal cortex underwent NFD [20,21]. Associated hippocampal neurons also degenerated with time, although there was no demonstration of a trans-synaptic transfer, as hippocampal NFD might also result from signal transduction related to receptors activation through Tau aggregation/oligomerisation. Moreover, the authors reported that hippocampal neurons also express mutated Tau species that may result from the non-specific activity of the neuropsin promoter [21,47]. To conclude, until now, only *in vivo* models of Tau capture [12,48] and/or Tau secretion have been developed [21].

The present study represents the first clear demonstration of the *in vivo* transfer of Tau proteins between anatomically connected neurons at distant brain (d > 10 mm). Within five months, Tau proteins spread to connected areas, such as the limbic and olfactory regions, where it appeared within secondary neurons. These V5-immunoreactive Tau species were primarily dephosphorylated (AT8 negative, ADx215 positive), suggesting that normal soluble Tau might be secreted and transferred into secondary neurons. Altogether these observations were further supported through studies in microfluidic chambers, where WT V5-Tau was identified, after 48 hours, in the axonal compartment in primary neurons. These results support previous data showing that Tau is secreted in a dephosphorylated state [41,43,49] and may activate muscarinic receptors (M1 and M3) leading to intracellular dysfunctions [40,49].

In this present study, we also demonstrated differential Tau spreading between WT Tau and mutant P301L Tau. Most studies of Tau propagation have used mutant forms, although a majority of tauopathies are sporadic and involve only the WT species and whether WT human Tau spreads as readily as the FTDP-17 Tau mutant is unknown. In the present model, Tau pathology associated with WT Tau was observed in brain areas connected to the IS, further supporting a trans-synaptic mechanism. In contrast, the Tau pathology induced through mutant P301L Tau protein remained near the LV injection site. Because P301L Tau mutant shows better nucleation and more rapid fibrillogenesis than does WT Tau [4], it is likely that the mutant protein might aggregate more readily, as observed using AT100-immunoreactivity, leaving fewer soluble species to migrate through axons. Moreover, consistent with the results of Caillierez et al. [22], neuronal death is higher with the P301L Tau mutant than with the WT Tau species. Such differential behaviour between the two Tau species also supports the *in vivo* specific trans-synaptic transfer of soluble Tau species rather than aggregates, as suggested using WT V5-Tau.

These findings also support the concept that Tau transmission occurs in sporadic diseases, such as AD, whereas Tau toxicity, leading to neuronal loss, more readily occurs in aggressive Tauopathies (FTDP-17) where all neurons have the mutant Tau, causing damage without spreading [5]. Taken together, the data derived from this *in vivo* model suggest that soluble/oligomeric forms of Tau protein drive spreading as suggested by Kayed’s group [14,50].

Finally, from data obtained from our *in vivo* model, one may argue that only a few secondary neurons displayed AT8-immunoreactivity and that it remains unclear whether this effect reflects the transfer of hyperphosphorylated Tau or a subsequent V5-Tau hyperphosphorylation occurring in secondary neurons. In any way, a few pathological Tau species are present in secondary neurons. Such observations are consistent with the slow kinetics of Tau spreading observed in sporadic Tauopathies. For in instance, in AD, the hierarchical spreading of Tau pathology, defined by the Braak stages, may last for 20 years [6]. Moreover, results generated from our model are of great relevance for future clinical investigations. Indeed, the discovery of distinct secreted/non secreted Tau species will define new perspectives in diagnosis of neurodegenerative diseases. Selection of patients at the beginning of the NFD spreading will then allowed the test of new therapeutics that would block this spreading and subsequently slow down the development of sporadic disease such as AD at the asymptomatic stages of disease. In this context for instance, Tau immunotherapy may be relevant for interfering with NFD in AD and related disorders referred to as Tauopathies. We showed previously than active immunotherapy allows for Tau clearance in the periphery and improves cognitive deficits promoted by Tau pathology in a well-defined mutant Tau mutant model [51]. More recently, passive immunotherapy further supports the targeting of extracellular Tau [52].

## Conclusions

In conclusion, independent of the mechanisms involved in Tau spreading, we demonstrated for the first time *in vivo* that the specific trans-synaptic transfer of Tau protein from degenerating neurons might lead to the preliminary steps of Tau pathology in secondary neurons. These data are of great interest considering that most tauopathies reflect the aggregation of Tau devoid of mutation. In the future, this model will facilitate a better understanding of Tau spread throughout the brain in sporadic tauopathies.

## Abbreviations

AcbSh: Accumbens nucleus shell; Amy: Amygdala; AOL: Anterior olfactory nucleus lateral part; Au1: Primary auditory cortex; AuD: Secondary auditory cortex dorsal area; AuV: Secondary auditory cortex ventral area; CA1-CA2-CA3: Fields of the hippocampus; cc: Corpus callosum; Den: Dorsal endopiriform nucleus; DG: Dendate gyrus; DP: Dorsal peduncular cortex; ec: External capsule; Ect: Ectorhinal cortex; Ent: Entorhinal cortex; fi: Fimbria of the hippocampus; fmi: Forceps minor of the corpus callosum; fmj: Forceps major of the corpus callosum; GrO: Granular layer of olfactory bulb; IL: Infralimbic cortex; Lent: Lateral entorhinal cortex; LO: Lateral orbital cortex; LSD: Lateral septal nucleus dorsal part; LSI: Lateral septal nucleus intermediate part; LSV: Lateral septal nucleus ventral part; PaS: Parasubiculum; Pir: Piriform cortex; PRh: Perirhinal cortex; PrL: Prelimbic cortex; PrS: Presubiculum; PtA: Parietal association cortex; S: Subiculum; SFi: Septofimbrial nucleus; TeA: Temporal association cortex; V2L: Secondary visual cortex lateral area; Ven: Ventral endopiriform nucleus.

## Competing interests

The authors declare no competing interests in this research.

## Authors’ contributions

MC and LB designed the study. SD, KL, RC, SB, NZ, CL, SC and MC performed the research. MC, SD, PH, NiD and LB analysed the data. NoD, PH, NiD, GA and JW contributed new reagents, materials and analyses. LB, MC, NiD and PH wrote the paper. All authors read and approved the final manuscript.

## Supplementary Material

Additional file 1 ***In-vitro *** **functionality of V5-hTau46**^WT^. (a) Schematic representation of the full-length 4R Tau isoform (2+3-10+) tagged with V5 epitope (GKPIPNPLLGLDST). HEK293 cells were infected with LVs encoding either V5-hTau46^WT^ or hTau46^WT^ and processed 48 hours later for immunofluorescence analysis using a rabbit polyclonal antibody to total V5-Tau (IF) (b), a polyclonal rabbit antibody to Tau C-Ter [23], which recognises the carboxyl terminal region of Tau, or a polyclonal rabbit antibody to GAPDH for biochemical assays (c) to demonstrate that the V5 tag did not alter transgene expression nor the electrophoretic mobility. Tau proteins were visualised using the corresponding secondary antibodies: Alexa Fluor-488 labelled goat anti-rabbit IgG antibody (green) for IF and peroxidase goat anti-Rabbit IgG antibody for biochemical assays. In (b), nuclei were labelled with DAPI are appear in blue. The scale bars are indicated on the figure. In (c), NI is related to the control non-infected cells. Click here for file

Additional file 2 **LVs particles are not diffusing in the microgrooves or trafficking along the microtubules.** (a) LVs were added to the somatodendritic compartment of a microfluidic device in the absence of cells. The flow was reversed to isolate compartments and then prevent viral diffusion. Viral RNA was extracted from somatodendritic and axonal compartments 5 min, 1, 12, 24 and 48 hours post-lentiviral delivery. cDNAs were generated and amplified using oligonucleotides specific to viral WPRE (540 bp). In the positive control, PCR was done from purified LVs batches and in the negative one without cDNA. (b) Coomassie Blue (2%) was added to the somatodendritic compartment in the absence of cells. The flow was reversed (upper panels) or not (lower panels) and the presence of colorant analysed 5 min, 1, 12, 24 and 48 later. The medium was recovered from both compartments and the optical density (O.D. 595 nm) analysed to determine the Coomassie Blue concentration. (c) Primary culture of embryonic rat cortical neurons seeded in the somatodendritic compartment was infected at DIV 7 with eGFP-LVs. The flow was then reversed and a second primary culture of embryonic rat cortical neurons was seeded in the axonal compartment. Forty-eight hours post-infection, cells were processed for visualizing eGFP fluorescence (green). The nuclei were counterstained with DAPI (blue). The scale bar is indicated on the figure. These data showed that LVs are not able to diffuse in the microgrooves compartment. It also showed that they are not trafficking along the microtubule. Tau transport in the microfluidic device is a specific mechanism since eGFP was not found associated to secondary connected neurons. Click here for file

Additional file 3 **Mutant-Tau-associated Tau pathology progression.** LVs encoding hTau46^P301L^ were bilaterally injected into the CA1 layer of rat brains (n = 5). Eight months later, the animals were sacrificed, and the whole brain was processed for immunohistochemical analysis using the AT8 antibody. In the middle upper panel, the total area positive for AT8 is represented from bregma -3.30 to bregma -7.47. The brain was virtually separated into three parts: bregma -2.30 to -4.30, bregma -4.52 to -6.04 and bregma -6.30 to -7.64. In each part, certain regions were selected to illustrate the Tau pathology (images on the left), and their location in the brain is shown on the right. The distribution of AT8 labelling (cell bodies (C) vs. neurites (N)) is presented in the tables. The scale bars are indicated on the figure. PhosphoTau (AT8 imunoreactivity) related to mutant Tau is restricted to the vicinity of the IS. Click here for file

Additional file 4 **WT Tau-associated Tau pathology progression.** LVs encoding hTau46^WT^ were bilaterally injected into the CA1 layer of rat brains (n = 5). Eight months later, the animals were sacrificed, and the whole brain is processed for immunohistochemical analyses using AT8. In the middle upper panel, the total AT8 immunopositive area is represented from bregma +5.20 to bregma -7.60. The brain was virtually separated into six parts: bregma +5.20 to +2.70, +2.20 to +1.40, bregma +1.20 to -1.40, bregma -1.80 to -4.30, bregma -4.52 to -6.04 and bregma -6.30 to -7.80. For each part, certain regions were selected to illustrate the Tau pathology (part left of the brain), and the total brain overlay is shown in the right part of the brain. The distribution of AT8 labelling (cellular bodies (C) vs. neurites (N)) is presented in the table. The scale bars are indicated on the figure. PhosphoTau (AT8 imunoreactivity) related to WT Tau is found all over the rat brain even in distant areas. Click here for file
