# Supplementary figures and images for: Neuron-to-neuron wild-type Tau protein transfer through a trans-synaptic mechanism: relevance to sporadic tauopathies
Source: Acta Neuropathol Commun. 2014 Jan 30;2:14. doi: 10.1186/2051-5960-2-14 (PMC3922636; doi:10.1186/2051-5960-2-14)

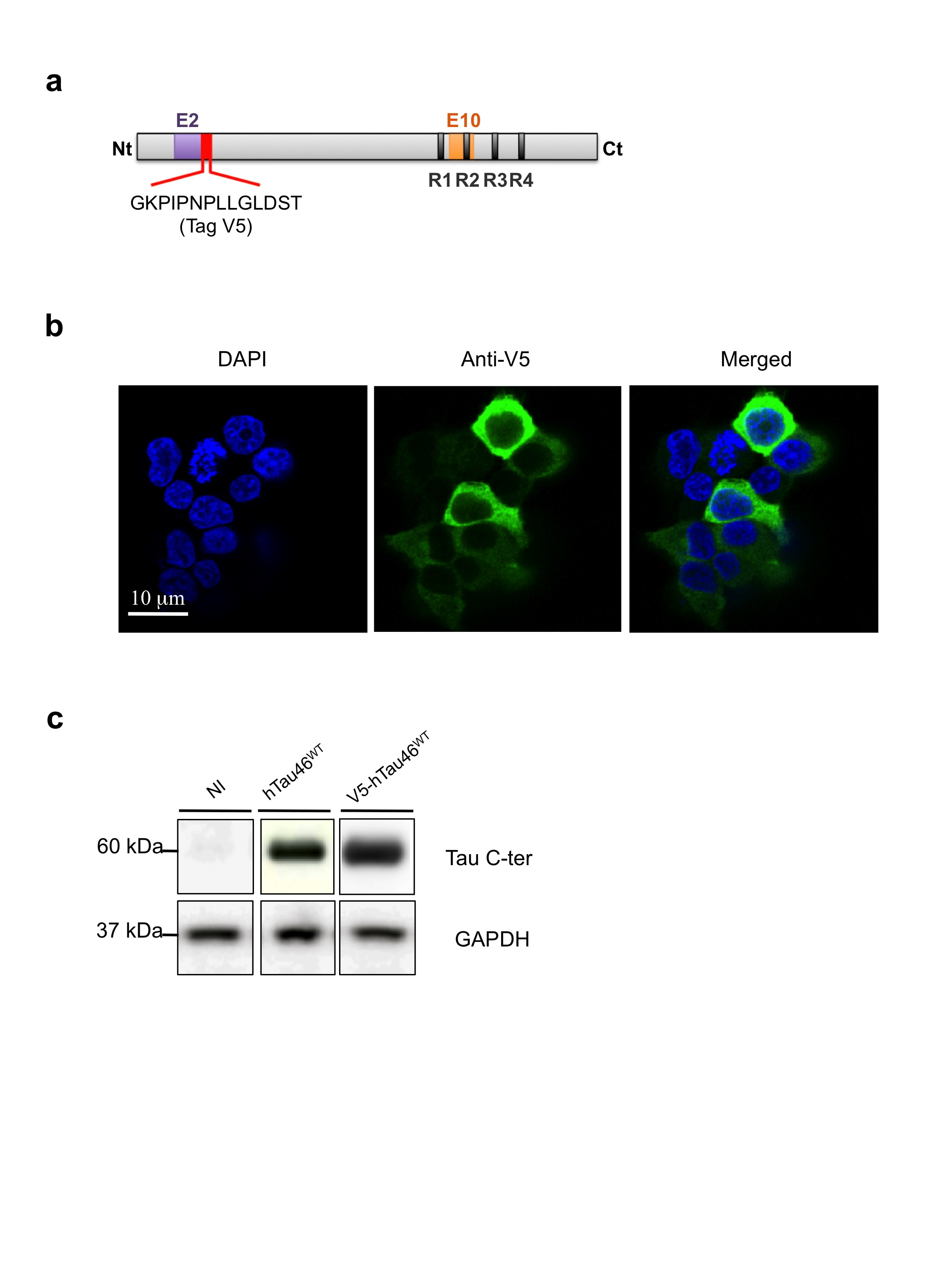

Supplement: Additional file 1 — In-vitro functionality of V5-hTau46WT. (a) Schematic representation of the full-length 4R Tau isoform (2+3-10+) tagged with V5 epitope (GKPIPNPLLGLDST). HEK293 cells were infected with LVs encoding either V5-hTau46WT or hTau46WT and processed 48 hours later for immunofluorescence analysis using a rabbit polyclonal antibody to total V5-Tau (IF) (b), a polyclonal rabbit antibody to Tau C-Ter [23], which recognises the carboxyl terminal region of Tau, or a polyclonal rabbit antibody to GAPDH for biochemical assays (c) to demonstrate that the V5 tag did not alter transgene expression nor the electrophoretic mobility. Tau proteins were visualised using the corresponding secondary antibodies: Alexa Fluor-488 labelled goat anti-rabbit IgG antibody (green) for IF and peroxidase goat anti-Rabbit IgG antibody for biochemical assays. In (b), nuclei were labelled with DAPI are appear in blue. The scale bars are indicated on the figure. In (c), NI is related to the control non-infected cells. [file 2051-5960-2-14-S1.jpeg]

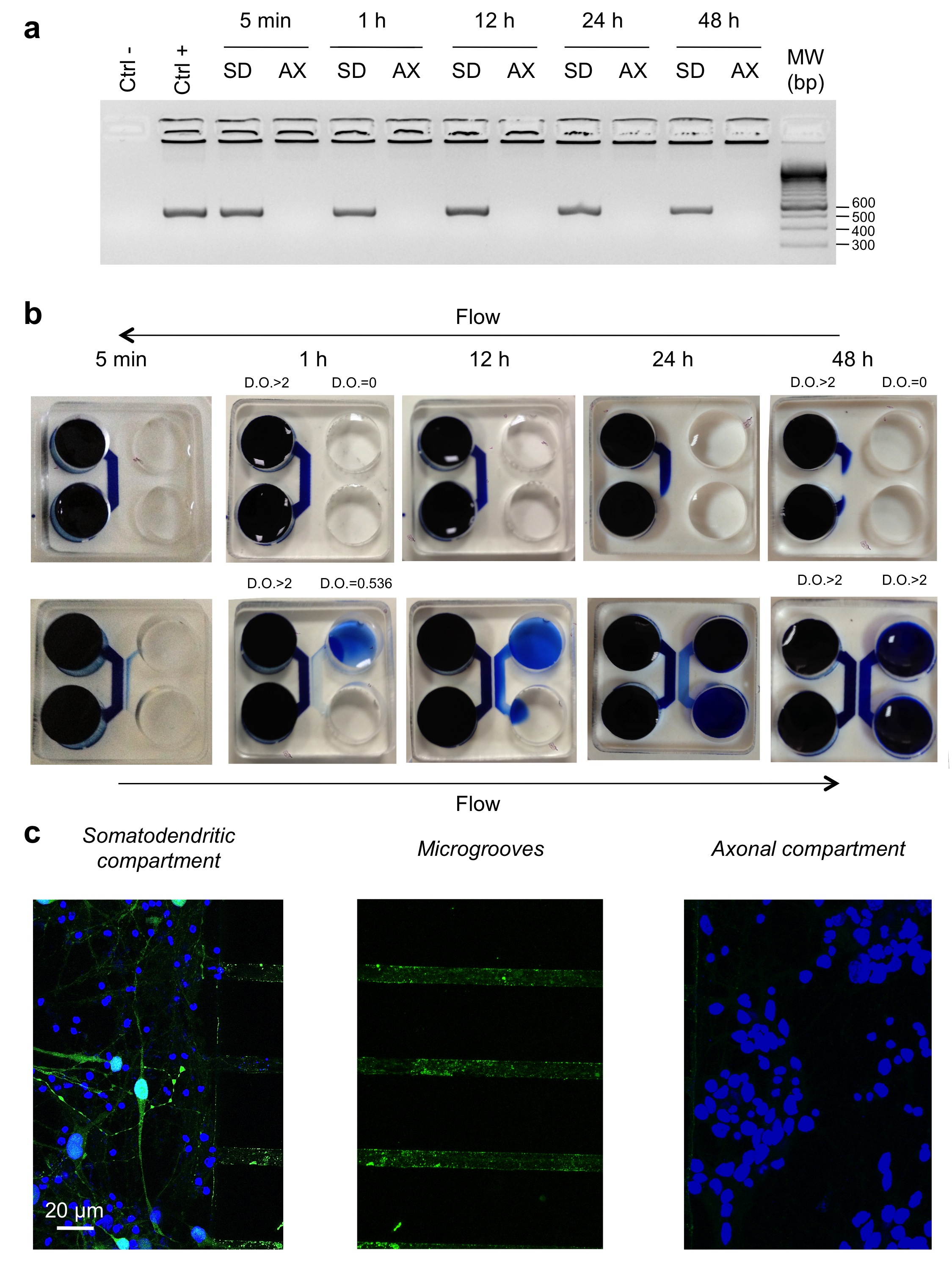

Supplement: Additional file 2 — LVs particles are not diffusing in the microgrooves or trafficking along the microtubules. (a) LVs were added to the somatodendritic compartment of a microfluidic device in the absence of cells. The flow was reversed to isolate compartments and then prevent viral diffusion. Viral RNA was extracted from somatodendritic and axonal compartments 5 min, 1, 12, 24 and 48 hours post-lentiviral delivery. cDNAs were generated and amplified using oligonucleotides specific to viral WPRE (540 bp). In the positive control, PCR was done from purified LVs batches and in the negative one without cDNA. (b) Coomassie Blue (2%) was added to the somatodendritic compartment in the absence of cells. The flow was reversed (upper panels) or not (lower panels) and the presence of colorant analysed 5 min, 1, 12, 24 and 48 later. The medium was recovered from both compartments and the optical density (O.D. 595 nm) analysed to determine the Coomassie Blue concentration. (c) Primary culture of embryonic rat cortical neurons seeded in the somatodendritic compartment was infected at DIV 7 with eGFP-LVs. The flow was then reversed and a second primary culture of embryonic rat cortical neurons was seeded in the axonal compartment. Forty-eight hours post-infection, cells were processed for visualizing eGFP fluorescence (green). The nuclei were counterstained with DAPI (blue). The scale bar is indicated on the figure. These data showed that LVs are not able to diffuse in the microgrooves compartment. It also showed that they are not trafficking along the microtubule. Tau transport in the microfluidic device is a specific mechanism since eGFP was not found associated to secondary connected neurons. [file 2051-5960-2-14-S2.jpeg]

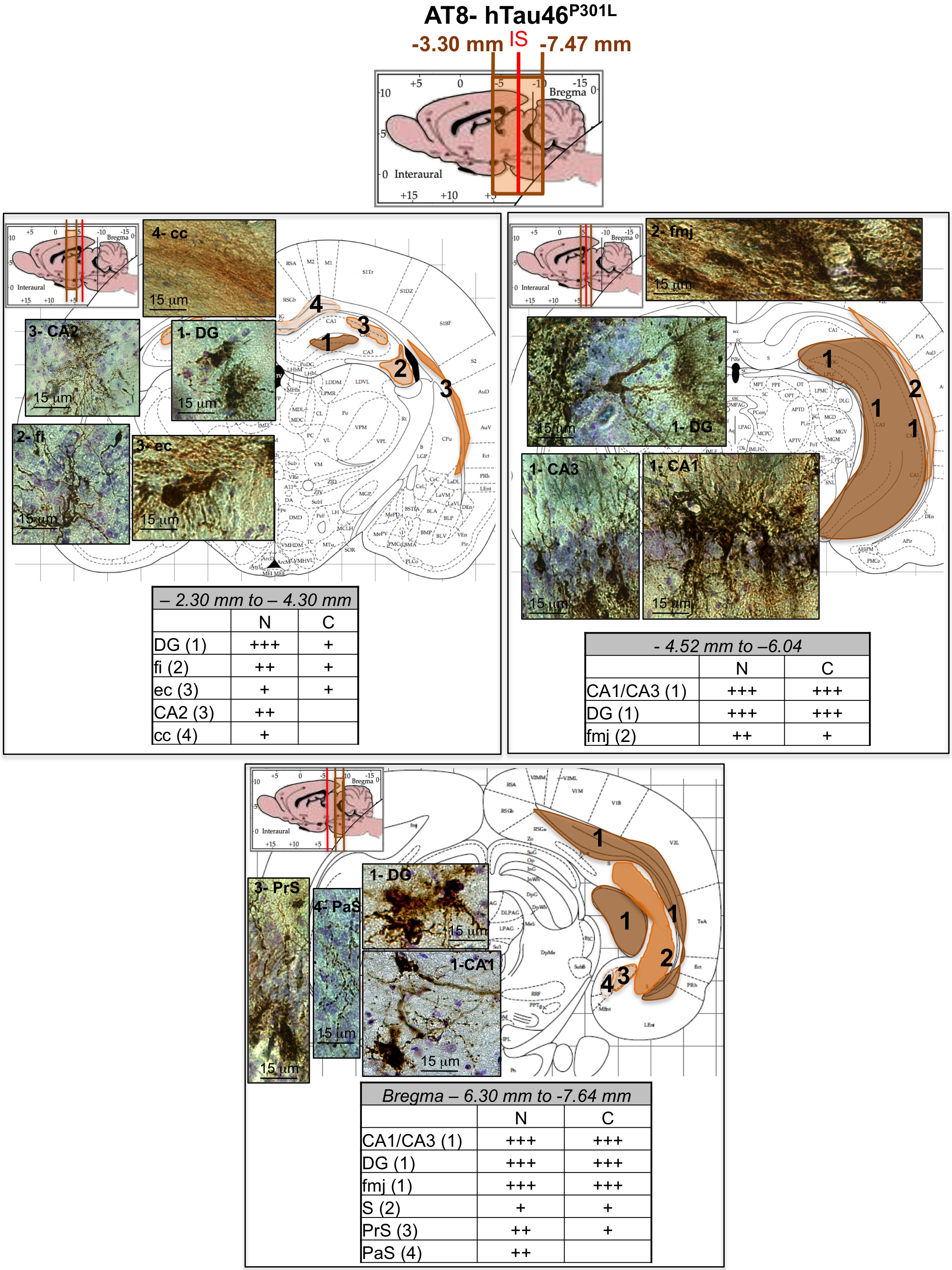

Supplement: Additional file 3 — Mutant-Tau-associated Tau pathology progression. LVs encoding hTau46P301L were bilaterally injected into the CA1 layer of rat brains (n = 5). Eight months later, the animals were sacrificed, and the whole brain was processed for immunohistochemical analysis using the AT8 antibody. In the middle upper panel, the total area positive for AT8 is represented from bregma -3.30 to bregma -7.47. The brain was virtually separated into three parts: bregma -2.30 to -4.30, bregma -4.52 to -6.04 and bregma -6.30 to -7.64. In each part, certain regions were selected to illustrate the Tau pathology (images on the left), and their location in the brain is shown on the right. The distribution of AT8 labelling (cell bodies (C) vs. neurites (N)) is presented in the tables. The scale bars are indicated on the figure. PhosphoTau (AT8 imunoreactivity) related to mutant Tau is restricted to the vicinity of the IS. [file 2051-5960-2-14-S3.jpeg]

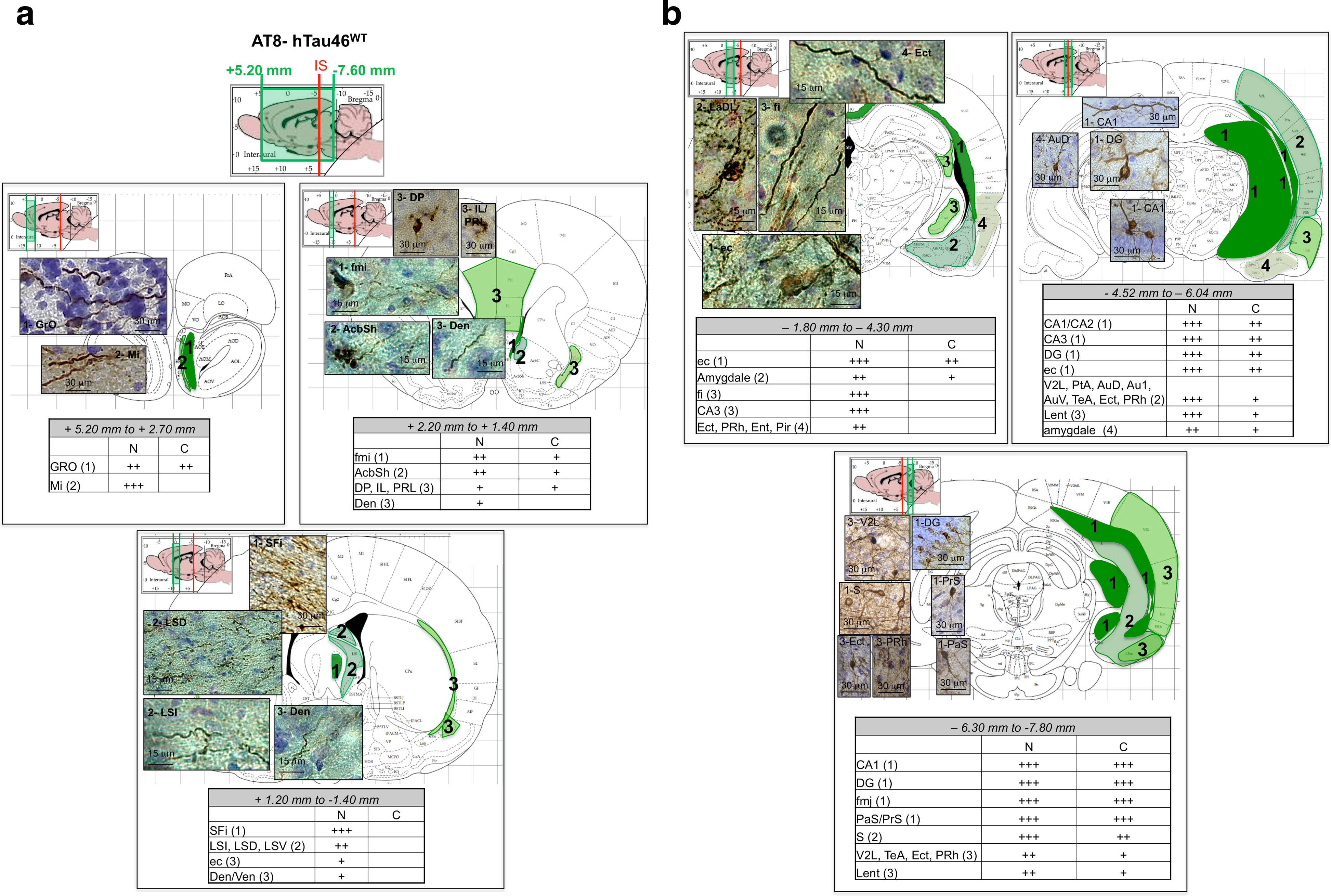

Supplement: Additional file 4 — WT Tau-associated Tau pathology progression. LVs encoding hTau46WT were bilaterally injected into the CA1 layer of rat brains (n = 5). Eight months later, the animals were sacrificed, and the whole brain is processed for immunohistochemical analyses using AT8. In the middle upper panel, the total AT8 immunopositive area is represented from bregma +5.20 to bregma -7.60. The brain was virtually separated into six parts: bregma +5.20 to +2.70, +2.20 to +1.40, bregma +1.20 to -1.40, bregma -1.80 to -4.30, bregma -4.52 to -6.04 and bregma -6.30 to -7.80. For each part, certain regions were selected to illustrate the Tau pathology (part left of the brain), and the total brain overlay is shown in the right part of the brain. The distribution of AT8 labelling (cellular bodies (C) vs. neurites (N)) is presented in the table. The scale bars are indicated on the figure. PhosphoTau (AT8 imunoreactivity) related to WT Tau is found all over the rat brain even in distant areas. [file 2051-5960-2-14-S4.jpeg]
